# The dual role of curcumin and ferulic acid in counteracting chemoresistance and cisplatin-induced ototoxicity

**DOI:** 10.1038/s41598-020-57965-0

**Published:** 2020-01-23

**Authors:** Fabiola Paciello, Anna Rita Fetoni, Daniele Mezzogori, Rolando Rolesi, Antonella Di Pino, Gaetano Paludetti, Claudio Grassi, Diana Troiani

**Affiliations:** 1 Department of Neuroscience, Università Cattolica del Sacro Cuore, Roma, Italia; 2 Fondazione Policlinico Universitario A. Gemelli IRCCS, Roma, Italia; 3 Department of Head and Neck Surgery, Università Cattolica del Sacro Cuore, Roma, Italia

**Keywords:** Oral cancer detection, Cochlea, Preclinical research

## Abstract

Platinum-based agents, such as cisplatin, form the mainstay of currently used chemotherapeutic regimens for several malignancies; however, the main limitations are chemoresistance and ototoxic side effects. In this study we used two different polyphenols, curcumin and ferulic acid as adjuvant chemotherapeutics evaluating (1) *in vivo* their antioxidant effects in protecting against cisplatin ototoxicity and (2) *in vitro* the transcription factors involved in tumor progression and cisplatin resistance. We reported that both polyphenols show antioxidant and oto-protective activity in the cochlea by up-regulating Nrf-2/HO-1 pathway and downregulating p53 phosphorylation. However, only curcumin is able to influence inflammatory pathways counteracting NF-κB activation. In human cancer cells, curcumin converts the anti-oxidant effect into a pro-oxidant and anti-inflammatory one. Curcumin exerts permissive and chemosensitive properties by targeting the cisplatin chemoresistant factors Nrf-2, NF-κB and STAT-3 phosphorylation. Ferulic acid shows a biphasic response: it is pro-oxidant at lower concentrations and anti-oxidant at higher concentrations promoting chemoresistance. Thus, polyphenols, mainly curcumin, targeting ROS-modulated pathways may be a promising tool for cancer therapy. Thanks to their biphasic activity of antioxidant in normal cells undergoing stressful conditions and pro-oxidant in cancer cells, these polyphenols probably engage an interplay among the key factors Nrf-2, NF-κB, STAT-3 and p53.

## Introduction

Cisplatin chemotherapy has been a mainstay of cancer treatment^1^. In general, cisplatin is considered a cytotoxic drug which kills cancer cells by the formation of intra- and inter-strand DNA crosslinks as well as cisplatin DNA adducts^2–4^. The molecular mechanisms of action are complex and involve multiple events that include induction of oxidative stress as characterized by reactive oxygen species (ROS) production and lipid peroxidation, inflammation by activating pro-inflammatory factors, induction of p53-dependent signaling pathways^5^. However, cisplatin chemotherapy is also associated with substantial side effects that include ototoxic damage^6–9^. Elevation of hearing threshold, mainly for the higher frequencies, has been reported in 22 to 75% of adult patients^10^ and 26 to 90% of pediatric patients^11^; it results in significant and permanent hearing loss that is especially debilitating in young children^11^. A growing body of research is dedicated to elucidate the molecular mechanisms implicated in cisplatin toxicity and resistance, including oxidative stress and inflammation^4,12–15^. Still, the molecular mechanisms underlying the enhanced sensitivity or resistance to cisplatin-induced apoptosis and cisplatin adverse effects are poorly understood. In the present research we considered that combination therapies of cisplatin with other drugs have become challenging in the treatment of human cancers to overcome drug resistance and reduce the undesirable side effects.

Currently, natural products or nutraceuticals are increasingly employed for adjunctive therapy of several malignances^16^ and a significant body of evidence has indicated that polyphenols may exert anti-cancer, chemosensitizing^17^ and cytoprotective effects against cisplatin toxicity in normal cells^18–20^. In addition, polyphenols modulate oxidative stress and inflammation^21–24^. This group of natural products found in plant-based foods, is highly diverse and contains several sub-groups chemically characterized as compounds with phenolic structural features. In addition to evidence of polyphenol direct antioxidant activity, there are indications that they may also act in ways beyond modulators of cell signaling as cardioprotectants, inhibitors of neurodegeneration and anticancer agents^23,25^. Among these, *Curcuma longa* extract, has been studied for its anti-inflammatory, antioxidant, anticancer and antiandrogenic effects^17,26^. The therapeutic benefits of curcumin have been demonstrated in multiple chronic diseases and, above all, in several cancers. Thus, curcumin represents a promising candidate as an effective anticancer drug to be used alone or in combination with other drugs^27^.

A strong antioxidant is also Ferulic acid (FA), widely studied even for its otoprotectant, antimicrobial, anti-arrhythmic, antithrombotic, antidiabetic and immuno-stimulant properties^25,28,29^. This phenolic acid gained attention for its potential role as an adjuvant therapy for several free radical-induced diseases, as ototoxicity, neurodegenerative disorders and cancer, considering that FA was proposed as a novel antioxidant compound endowed with a strong cytoprotective activity due to both the ability to scavenge free radicals and activate cell stress response^28^.

In spite of the increasing efforts to study properties and effectiveness of curcumin and FA in the model of oxidative stress-related diseases, there are still several issues to be addressed as regard to their potency and specificity in cancer. Thus and with the main focus addressed to the analysis of cisplatin side effects and resistance, we used curcumin and FA as adjuvant to cisplatin in an *in vivo* model of otototoxicity and in an *in vitro* model of oral cell carcinoma, a common aggressive malignancy that is refractory to most therapeutic interventions. We studied respectively, the relationship between cytotoxicity, oxidative stress and inflammation and the possible implications among a) Nrf-2 that controls a cellular defensive response^30^, b) NF-κB a master regulator of the inflammatory process, responsible for the widespread systemic inflammatory process^31^ and for tumor resistance^32^ and c) p53 that mediates the induction of apoptosis^33^.

## Results

### *In vivo* experiments

#### Auditory function evaluation

**Figure 1:**
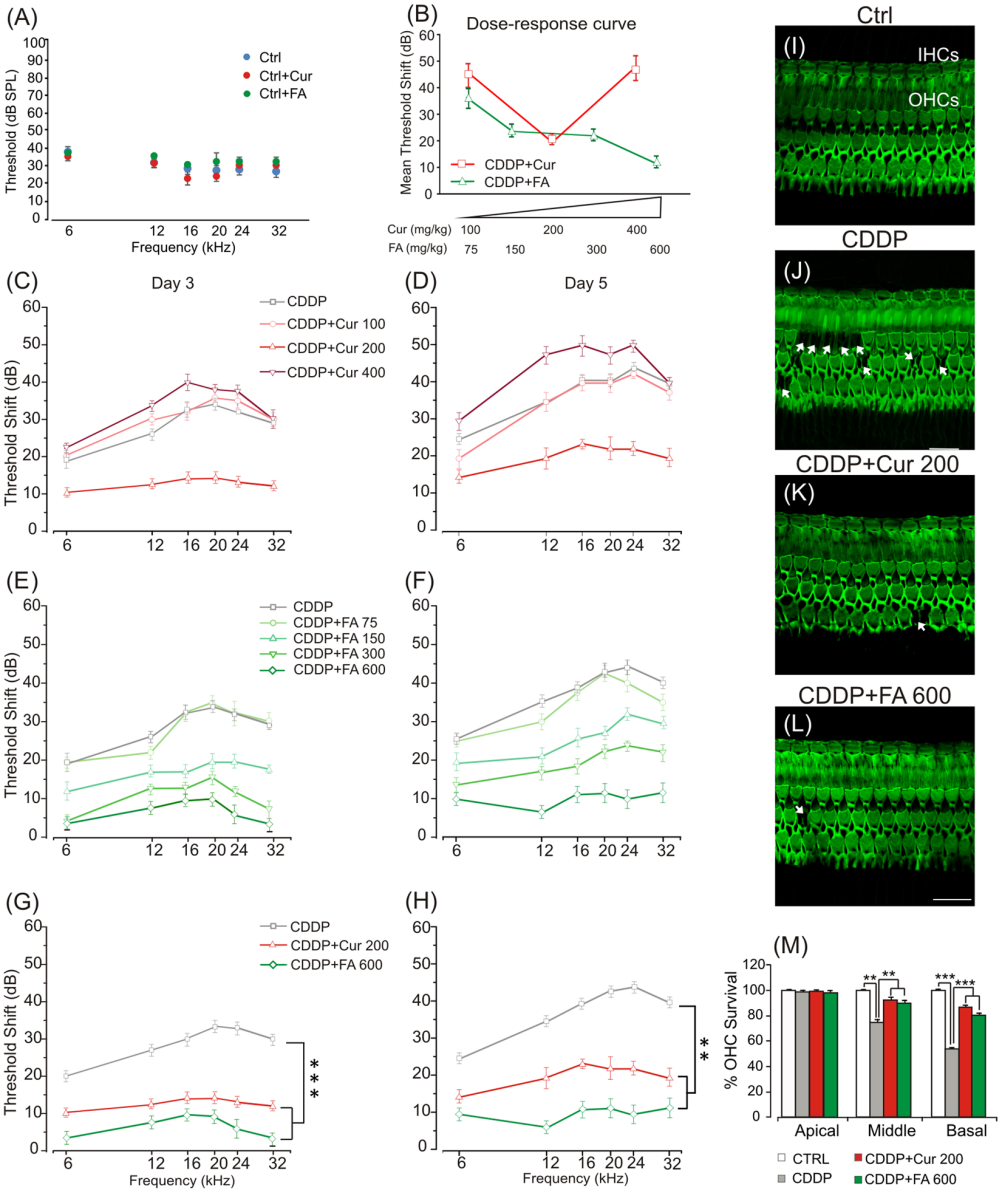
Curcumin and FA protect against functional and morphological damage induced by cisplatin in the cochlea. (**A**) Graph shows auditory threshold values (means ± SEM) in normal-hearing animals treated with the most effective dose of curcumin (200 mg/kg) and FA (600 mg/kg). (**B**) Graph (means ± SEM) shows dose-response curves (mean ABR threshold shift values across all tested frequencies) for curcumin (red) and FA (green) treatment. (**C–H**) Graphs show threshold shift values (means ± SEM) in animals treated with cisplatin (CDDP group) and cisplatin + curcumin (CDDP + Curr group; **C**,**D**) or cisplatin + ferulic acid (CDDP + FA group; **E**,**F**) at different doses. The effect of cisplatin and curcumin or FA administration at the most effective dose is shown in **G** and **H**. Curcumin at a dose of 200 mg/kg protects against hearing loss of about 15–20 dB both 3 (**C,G**) and 5 (**D,H**) days after cisplatin treatment. FA administration shows a dose-dependent protective effect: the most effective dose (600 mg/kg) induces a protection of about 20–25 dB 3 (**E,G**) and 5 (**F,H**) days after cisplatin treatment. (**I–L**) Representative images of F-Actin staining in surface preparations of the organ of Corti. A typical distribution of one row of inner hair cells (IHCs) and of the three rows of outer hair cells (OHCs) are shown in Ctrl group (**I**). Cisplatin administration causes OHC loss (dark spots and phalangeal scars, **J**). Curcumin (**K**) and FA (**L**) administration protects against OHC death. Arrows indicate missing cells. Scale bar: 20 μm. (**M**) Cochleogram shows percentage of OHC survival in the cochlear turns in all experimental groups. Asterisks indicate significant differences between groups (**p < 0,01; ***p < 0,001). Morphological data are representative of three independent experiments, n = 6 cochleae per group.

To assess the most effective curcumin and FA doses against cisplatin-induced ototoxicity, we constructed dose/response curves by recording Auditory Brainstem Responses (ABRs) in all animals before (day 0), 3 and 5 days after cisplatin treatment (Fig. 1C–F). Baseline ABR thresholds did not differ among the experimental groups. Cisplatin administration induced a threshold elevation of about 35–40 dB at days 3 and 5 respectively (Fig. C–H). Treatment with curcumin 200 mg/kg decreased cisplatin ototoxicity of about 15–20 dB at the same time points (Fig. 1C,D,G,H). However, the lower dose of curcumin (100 mg/kg) had no effect and the higher dose (400 mg/kg) worsened, at day 5, the cisplatin damage (Fig. 1C,D). FA administration showed a dose-dependent protective effect against cisplatin ototoxicity: the lowest dose of 75 mg/kg had no protective effect, whereas starting from the dose of 150 mg/kg, FA attenuated cisplatin-induced hearing loss (Fig. 1E,F). The most effective dose was 600 mg/kg, attenuating cisplatin ototoxicity of about 20–25 dB (Fig. 1E,F,G,H). Notably, ABR thresholds did not differ among control animals and animals treated with the most effective curcumin (200 mg/kg) or FA (600 mg/kg) dosage (Fig. 1A). Taken together these data demonstrate that FA showed a dose-dependent effect on hearing function, decreasing threshold shift values by increasing the dosage (Fig. 1B). On the other hand, the mid dose of 200 mg/kg of curcumin significantly attenuated hearing loss caused by cisplatin (Fig. 1B,G,H) indicating that this molecule shows an hormetic effect, exhibiting a biphasic response to increasing doses.

#### Morphological evaluations in the cochlea

To further characterize cochlear damage we performed F-Actin staining to determine hair cell loss. Figure 1, right panel, shows cochleograms of Ctrl, CDDP, CDDP + Cur and CDDP + FA groups at the most protective doses (200 mg/kg for curcumin and 600 mg/kg for FA) evaluated 5 days after cisplatin treatment. Cisplatin administration induced a marked hair cell (HC) loss with disappearance of both cuticular plane and hair bundle, mostly in the first and second outer hair cell (OHC) rows of middle and basal turns (Fig. 1J). Moreover, OHC count showed approximately 55% of hair cell survival in basal turn and about 75% in middle turn in cisplatin group as compared with Ctrl (Fig. 1M). In the CDDP + Cur 200 group, the survival rate reached about 85% and 90% in the same regions of basal and middle turns, respectively (Fig. 1K,M). Similarly to curcumin, FA administered at a dose of 600 mg/kg, showed protective effects against cisplatin-induced OHC loss: cochleogram data indicate a cell survival of about 90% in the middle turn and a slight lower protection on the basal turn (about 80% of cell survival rate) although not statistically significant as compared to curcumin effect in the basal region (Fig. 1L,M).

#### Polyphenols induce Nrf-2/HO-1 pathway activation in the cochlea

**Figure 2:**
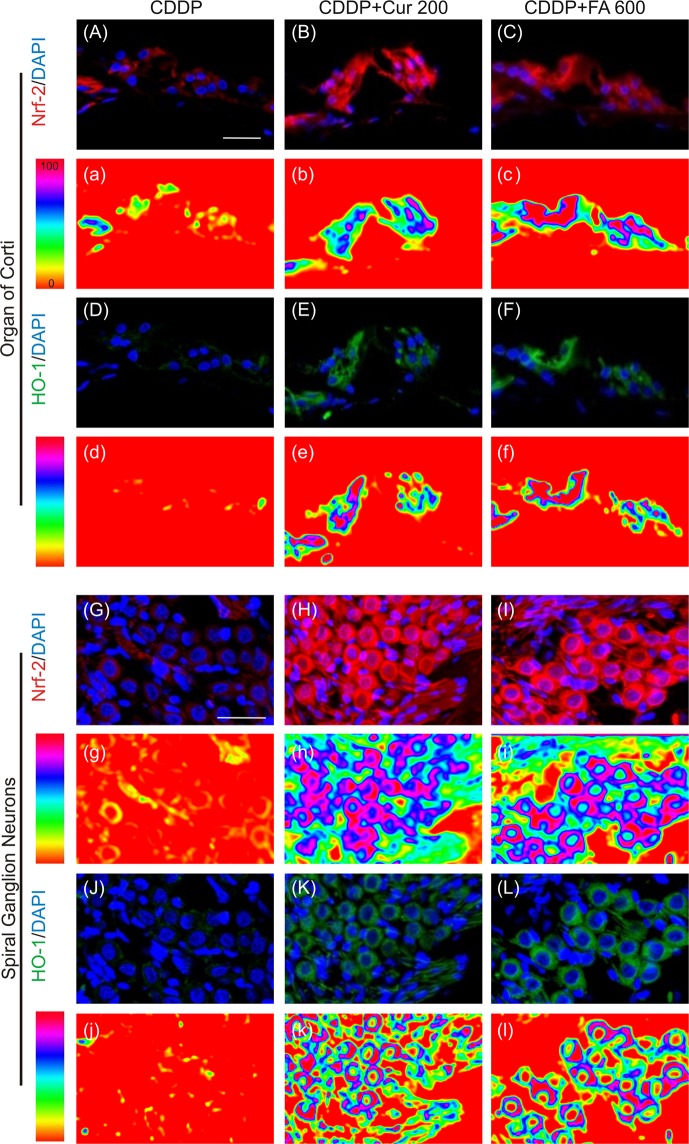
Curcumin and ferulic acid potentiate the endogenous antioxidant response against cisplatin ototoxicity. Representative images of the organ of Corti (**A–F**) and SGNs (**G–L**) double-labelled with antibody against Nrf-2 (red fluorescence) and HO-1 (green fluorescence) and stained with DAPI (blue fluorescence). The distribution of Nrf-2 and HO-1 fluorescence signals in the organ of Corti (d-f) and SGNs (g-l) are shown in a pseudo-color rainbow scale. Cisplatin treatment induces a slight Nrf-2/HO-1 activation, both in the organ of Corti (**A,D**) and in SGNs (**G,J**). Curcumin and FA supplementation (**B,H** and **C,I**) induces an increase of Nrf-2 nuclear translocation, paralleled by an increase of HO-1 expression, both in the organ of Corti and in SGNs, potentiating the endogenous antioxidant responses. Scale bar: 30 μm. SGNs: spiral ganglion neurons. Data are representative of three independent experiments, n = 6 cochleae per group.

To explore the antioxidant adaptive mechanism underlying otoprotection we performed immunofluorescence analyses for Nrf-2/HO-1 activation in cochlear cryosections of Ctrl, CDDP, CDDP + Cur 200 and CDDP + FA 600 groups. Figure 2 shows representative images of Nrf-2 and HO-1 immunostainings of the organ of Corti (Fig. 2A–F) and spiral ganglion neurons (SGNs; Fig. 2G–I). In control specimens, Nrf-2 and HO-1 labelling was faint and slightly displaced in the cytosol (data not shown), consistent with our previously published results^18^. In both organ of Corti and SGNs of cisplatin specimens, Nrf-2 (Fig. 2A,G) and HO-1 (Fig. 2D,J) labelling was mainly localized in the cytoplasm (Fig. 2a,g,d,j). Treatment with curcumin 200 mg/kg induced an increase of both expression and nuclear translocation of Nrf-2 (Fig. 2B,b,H,h). In these specimens, high Nrf-2 fluorescence signal was detected not only in the cytoplasm but also in the nucleus, both in OHCs (Fig. 2B,b) and in SGNs (Fig. 2H,h). In parallel, in the same samples, we observed an over-expression of HO-1 both in the organ of Corti (Fig. 2E,e) and in SGNs (Fig. 2K,k), confined inside the cytoplasm. The data on curcumin upregulation of Nrf-2/HO-1 pathway are in agreement with previous results^18^. Interestingly, our results showed that FA modulated the same antioxidant pathway targeted by curcumin. In fact, FA treatment (600 mg/kg) induced an enhancement of Nrf-2 expression and its nuclear translocation both in the organ of Corti and in SGNs (Fig. C,I) matched by an up-regulation of HO-1 protein expression in the same structures (Fig. 2F,L). Collectively, our data indicate that curcumin and FA produced a similar antioxidant effect against cisplatin ototoxicity, sharing the same activation of Nrf-2/HO-1 pathway, probably to face the redox imbalance caused by cisplatin administration.

#### NF-κB expression is reduced by curcumin administration in the cochlea

**Figure 3:**
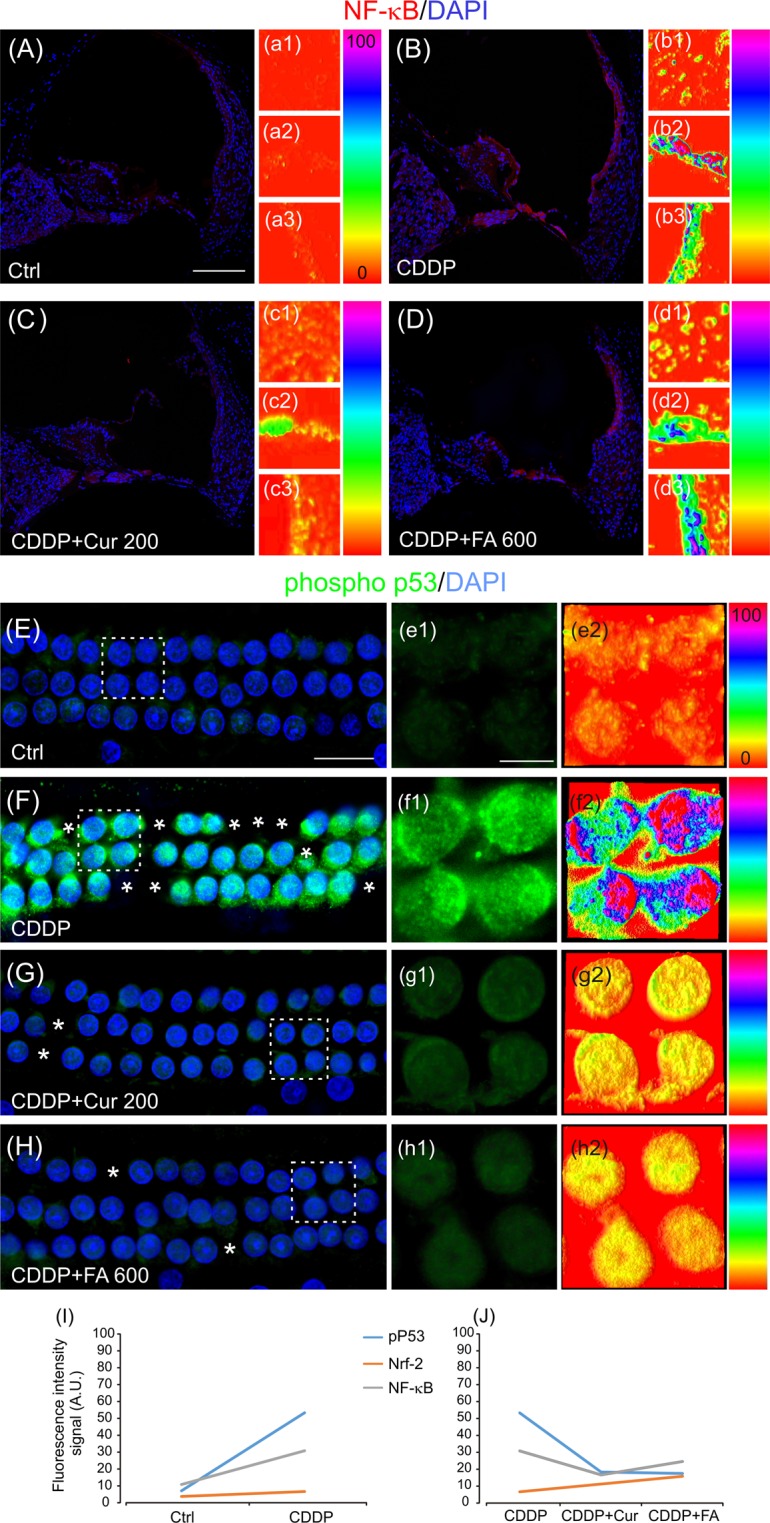
Anti-inflammatory and anti-apoptotic activity of polyphenols in cisplatin-induced ototoxicity. (**A–D**) Representative images of cochlear cryosections stained with NF-κB (red fluorescence) and DAPI (blue fluorescence) in all experimental groups. The distribution of fluorescence signal, analyzed in a pseudo-color rainbow scale, is shown for the principal cochlear structures: spiral ganglion neurons (a1–d1), the organ of Corti (a2–d2) and *stria vascularis* (a3–d3). In Ctrl samples a weak NF-κB fluorescence signal is observed (**A**). Cisplatin treatment induces an increase of NF-κB expression, mainly in the organ of Corti and *stria vascularis* (**B**). Curcumin administration (**C**) reduces significantly NF-κB fluorescence, whereas FA (**D**) is ineffective in attenuating NF-κB activation induced by cisplatin. Scale bar: 100 μm. (**E–H**) Representative images of surface preparations of the organ of Corti showing three rows of OHCs labeled with phospho p53 (green fluorescence) and DAPI stained (blue fluorescence) in all experimental groups. Dotted squares indicate high magnifications shown in e1–h1 respectively. e2–h2 show the distribution of fluorescence signal in a pseudo-color rainbow scale. Curcumin at a dose of 200 mg/kg (**G**) and FA at a dose of 600 mg/kg (**H**) reduce significantly the increase of p53 phosphorylation induced by cisplatin. Asterisks in F–H indicate missing cells. Scale bar: E, 20 μm; e1, 10 μm. Data are representative of three independent experiments, n = 6 cochleae per group. (**I–J**) Graphs show the trend of pP53, Nrf-2 and NF-κB activation (fluorescence intensity signal, arbitrary units, A. U.) in cochlear cells after cisplatin (I) and polyphenols treatment (J).

We performed immunofluorescence analysis of NF-κB in cochlear cryosections to establish the possible anti-inflammatory effect of polyphenols. Figure 3 shows NF-κB expression in cochlear cryosections at day 5 after cisplatin treatment in all experimental groups. Fluorescence was faint in controls (Fig. 3A), but increased markedly in the organ of Corti and *stria vascularis* after cisplatin administration, as also indicated by fluorescence intensity spectrum (Figs. 3B,b1–b3). Namely, cisplatin treatment shows a pro-inflammatory effect in the cochlea by increasing NF-κB expression. Curcumin reduced significantly NF-κB in the organ of Corti, SGNs and *stria vascularis* and fluorescence signal reversed almost to control level (Figs. 3C,c1–c3). Interestingly, after FA treatment, the increase of NF-κB fluorescence induced by cisplatin remained stable in the organ of Corti and in the *stria vascularis* and only a slight fluorescence decrease was observed in SGNs (Figs. 3D,d1–d3). Results in this model of ototoxicity suggest that polyphenol properties are however different, indicating that only curcumin, but not FA, produced an anti-inflammatory effect.

#### Curcumin and ferulic acid reduce p53 phosphorylation in the cochlea

To investigate the mechanisms of hair cell death, we analyzed the role of p53, considering that p53 status is an important determinant of cisplatin sensitivity. Figure 3, lower panel, shows immunofluorescence for p53 phosphorylation (phospho S15) in surface preparations of the organ of Corti, at day 5 after cisplatin treatment in Ctrl, CDDP, CDDP + Cur 200 and CDDP + FA 600 groups. A slight labeling for phospho p53 was found in control samples (Fig. 3E), as confirmed by fluorescence intensity spectrum which showed a low fluorescence signal both in the cytoplasm and nucleus (Fig. 3e1,e2). Cisplatin treatment increased p53 phosphorylation, specifically in the nucleus, as indicated by fluorescence intensity (Figs. 3F,f1,f2). Notably, the antioxidant treatment with curcumin or FA decreased p53 phosphorylation and nuclear fluorescence signal was reduced (Figs. 3G,H,g,h1,g,h2). These data indicate that the cochlear p53 upregulation induced by cisplatin is reduced by polyphenol treatment: in fact, both curcumin and FA show anti-apoptotic effects at the effective dosage. Quantitative analysis of fluorescence signal intensity confirmed these data (Fig. 3I,J).

### *In vitro* Experiments

#### Polyphenols increase cisplatin-induced cytotoxicity in cancer cells

**Figure 4:**
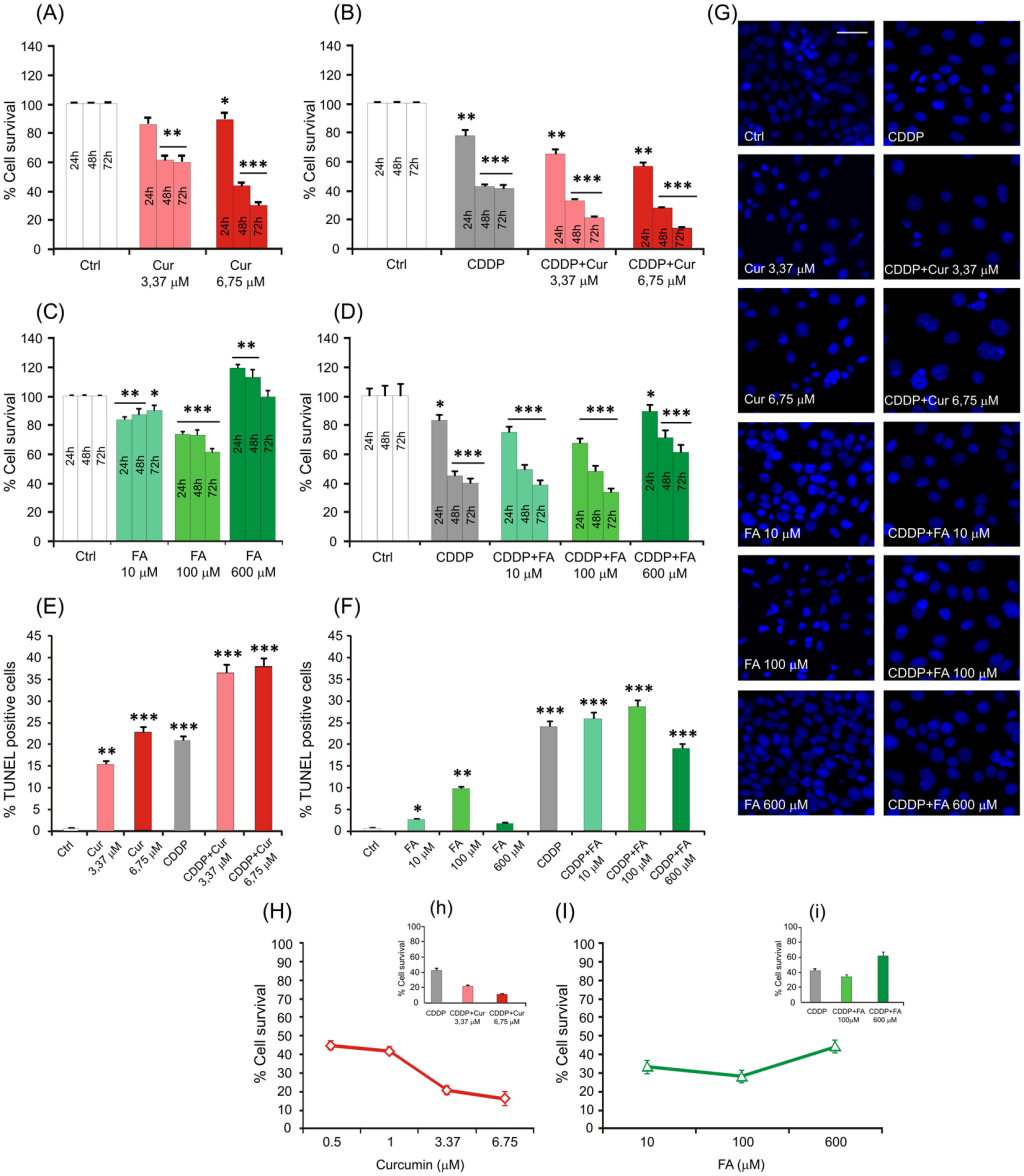
Polyphenols increase cisplatin-induced cytotoxicity in cancer cells. (**A**–**D**) Graphs (mean ± SEM) show percentage of cell survival in PECA/PJ-15 treated with different doses of curcumin (**A**), cisplatin + curcumin (**B**), FA at different doses (**C**) and cisplatin + FA (**D**) at 24, 48 and 72 h after treatment. Curcumin shows a cytotoxic effect *per se* in tumor cells (**A**) and it has an adjuvant effect, potentiating the cytotoxic effect of cisplatin (**B**). FA shows a slight anti-tumoral effect at the dose of 100 μM, whereas at high dose (600 µM) a pro-proliferative effect is observed (**C**,**D**). (**E**–**F**) Histograms show percentage of TUNEL-positive cells treated with cisplatin and/or curcumin (**E**) and cisplatin and/or FA (**F**). (**G**) Representative images of DAPI staining in cancer cells for the different polyphenols and doses used. (**H–I**) Dose-response curves showing the percentage of cancer cell survival (mean ± SEM) at increasing curcumin (**H**) or FA (**I**) concentrations. Asterisks refers to significant differences compared to control condition (*p < 0,05; **p < 0,001; ***p < 0,0001). Scale bar: 40 μm. Data are representative of three independent experiments, each count was performed on 10 fields randomly selected for each experimental condition and each time point.

In order to determine the cytotoxicity of cisplatin and to compare curcumin and FA treatment effects in cancer cells, the survival rate and apoptosis of PE/CA-PJ15 cells were evaluated at 24, 48 and 72 h after treatment. Cisplatin monotherapy induced a cell loss of ~20% at 24 h, increasing in later time points to ~55% and 60% after 48 and 72 h of incubation, respectively (Fig. 4B,D). Moreover, the number of TUNEL-positive cells, evaluated 72 h after treatment, increased of about 20% (Fig. 4E,F) confirming the cytotoxic and pro-apoptotic effect of cisplatin. Curcumin monotherapy induced a cytotoxic effect in a dose-dependent manner (Fig. 4A). As shown previously^18^, 0.5 µM induced a slight cell loss that progressed over time to reach ~20% of cell loss after 48 and 72 h of incubation; 1.0 µM showed a similar effect, ~20% and 25% of cell loss at 48 and 72 h, respectively (data not shown). Curcumin treatment at the concentration of 3.37 µM caused ~40% of cell loss at 72 h and 6.75 µM caused ~60–70% of cell loss at 48 and 72 h, respectively (Fig. 4A). Similarly, the percentage of TUNEL-positive cells increased in a dose-dependent manner, reaching about 15–20% at high curcumin doses (3.37 and 6.75 µM, Fig. 4E).

Conversely, FA administration induced a slight cell loss of about 20% when administered at 10 µM (Fig. 4C). At the dose of 100 µM, FA showed a higher cytotoxic effect: we observed a cell death of about 25% at 24 h that remained stable at 48 h after treatment and increased of about 40% after 72 h of incubation (Fig. 4C). TUNEL assay showed a small anti-apoptotic effect of FA: the percentage of TUNEL-positive cells was less than 5% at the dose of 10 µM and ~10% at the dose of 100 µM. Interestingly, increasing the dose of FA, we found an opposite effect: when administered at 600 µM, the FA showed a pro-proliferative effect and the number of viable cells in the first 48 h after administration increased of about 20% with respect to control condition (Fig. 4C). TUNEL assay confirmed these data, showing less than 5% of apoptotic cells after treatment with 600 µM FA (Fig. 4F).

Furthermore, we analyzed the effect of the combined exposure of cisplatin + curcumin or cisplatin + FA on cancer cells. The adjuvant curcumin doses of 0.5 and 1.0 µM determined cell loss of ~30%, 40% and 60% at 24, 48 and 72 h, respectively (data not shown). This effect increased at the dose of 3.37 µM causing 70% and 80% of cell death after 48 and 72 h, respectively (Fig. 4B). A further increase of cell death was observed for the concentration of 6.75 µM, mainly 72 h after treatment (10–15% cell survival; Fig. 4B). When administered in conjunction with cisplatin, FA at low and medium doses (10 and 100 µM) did not improve significantly the cytotoxic effect of cisplatin; conversely, the higher dose of FA (600 µM) showed protective effects against cisplatin cytotoxicity, increasing cell survival of about 10% at 24 h and 20% at 48 h that was stable until 72 h, with respect to cisplatin treated cells (Fig. 4D). TUNEL assay confirmed these results: curcumin potentiated the effect of cisplatin in a dose-dependent manner, increasing the number of apoptotic cells (Fig. 4E), whereas FA (600 µM) counteracted the pro-apoptotic effect of cisplatin and the percentage of TUNEL-positive cells decreased of about 10% (Fig. 4F).

Namely, curcumin supplementation increased the cytototoxic effect of cisplatin in a dose-dependent manner (Fig. 4H,h), whereas FA adjuvant administration, at a concentration of 100 µM, induced a slight increase of cell death (Fig. 4I,i). Conversely, the higher dose (600 µM) showed a protective effect *versus* cisplatin cytotoxicity, by enhancing the number of viable cells (Fig. 4I,i). Interestingly, FA acts as an hormetin: at low and medium doses it shows anti-tumoral effects, whereas at high dose it potentiates cell survival and proliferation (Fig. 4I).

#### Polyphenols modulate Nrf-2 nuclear translocation in cancer cells

**Figure 5:**
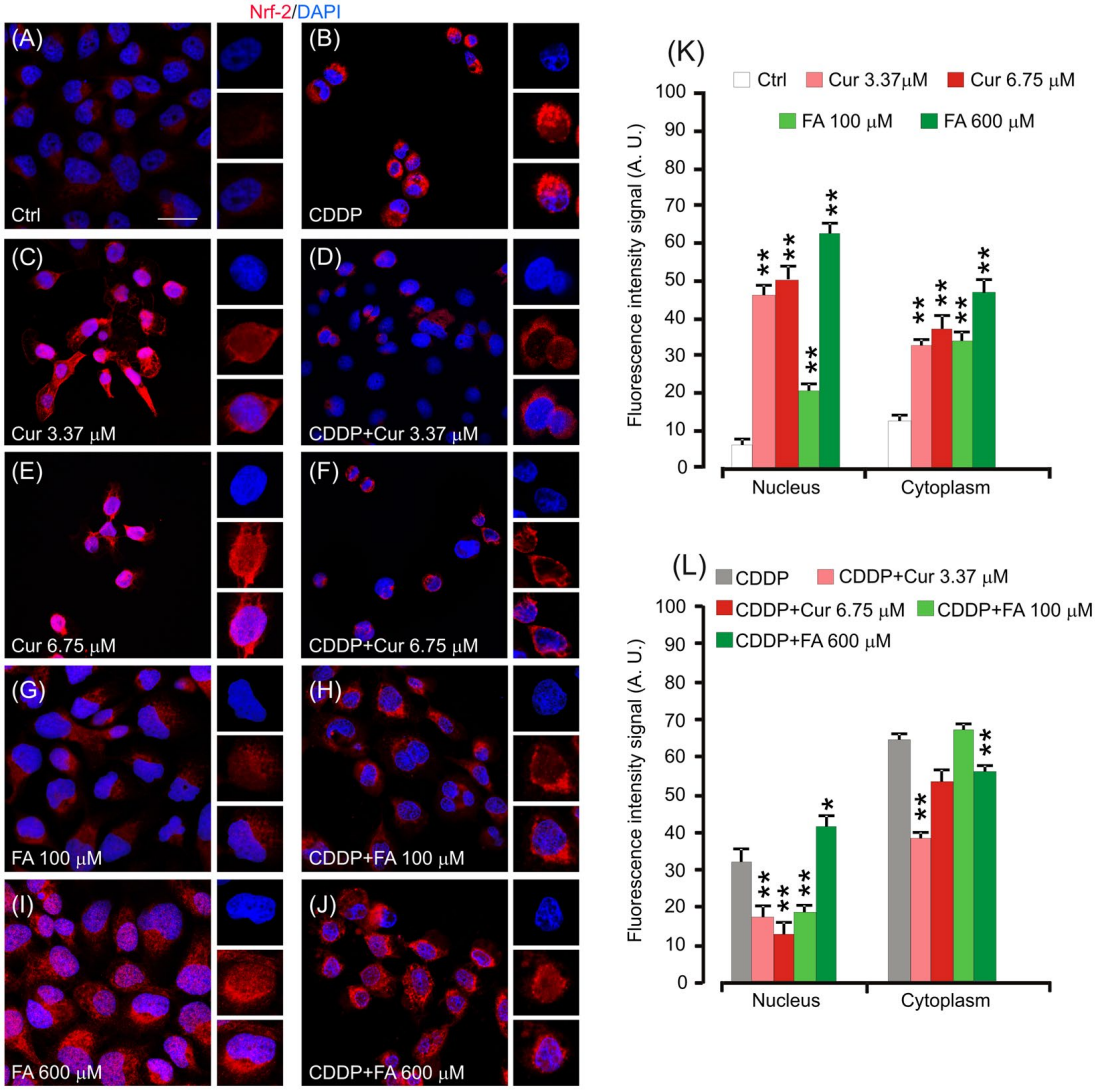
Polyphenols show antioxidant properties in monotherapy and pro-oxidant effects when administered in conjunction with cisplatin. (**A**–**J**): Representative images of PECA–PJ15 immunolabelled with antibody against Nrf–2 (red fluorescence) and stained with DAPI (blue fluorescence). High magnifications in boxes show nuclear *vs* cytosolic fluorescence localization. (**K**,**L)**: Histograms (mean ± SEM) show fluorescence intensity signal quantification (A.U., arbitrary units) in the nucleus or in the cytoplasm of treated cells. Asterisks refers to significant differences compared to control condition in **K** and to cisplatin condition in **L** (*p < 0,05; **p < 0,01). Scale bar: 20 μm. Data are representative of three independent experiments, each count was performed on 10 fields randomly selected for each experimental condition and each time point.

To characterize the cytotoxic effect on cancer cells exerted by curcumin and FA in adjuvant to cisplatin we investigated Nrf-2 activation. Immunofluorescence data revealed a slight Nrf-2 labelling in control condition (Fig. 5A,K). Cisplatin monotherapy induced a marked raise of Nrf-2 labelling (Fig. 5B), primarily cytoplasmic as indicated by fluorescence signal quantification (Fig. 5L), probably as a consequence of cell altered redox-status induced by cisplatin. PE/CA-PJ15 exposed to different doses of curcumin showed an increase of nuclear Nrf-2 fluorescence, in a dose-dependent manner (Fig. 5C,E,K), indicating the antioxidant effect of curcumin monotherapy. Conversely, the adjuvant curcumin doses to cisplatin caused an increase in Nrf-2 activation that, however, remained restricted inside the cytoplasm, without translocation into the nucleus (Fig. 5D,F,L), indicating, in this case, a pro-oxidant effect of the molecule that could be responsible for the increased cell death observed in the combined therapy. By comparing the effects of the two polyphenols, our results show that, similarly to curcumin, FA administration showed antioxidant effects, inducing an increase of Nrf-2 expression and translocation into the nucleus, in a dose-dependent manner (Fig. 5G,I,K). Interestingly, in the combined treatment with cisplatin, cells treated with 100 µM FA showed a Nrf-2 expression that did not significantly differ from what observed in cisplatin monotherapy (Fig. 5H,L) and the fluorescent signal was localized mainly in the cytoplasm. On the contrary, cells treated with the higher dose of FA (600 µM) showed an increase of nuclear Nrf-2 fluorescent signal with respect to cisplatin monotherapy (Fig. 5J,L). These data indicate that, curcumin shows pro-oxidant dose-dependent effects, probably counteracting cisplatin resistance related to Nrf-2 activation, whereas FA exhibits pro-oxidant properties only at 100 µM and exerts antioxidant effect at a higher dose. Taken together, our results indicate that the polyphenols, used as adjuvant to cisplatin, induce a remarkable pro-oxidant activity indicative of a possible anti-chemoresistance effect, even depending on the dosage for FA.

#### Curcumin modulates NF-κB expression in cancer cells

**Figure 6:**
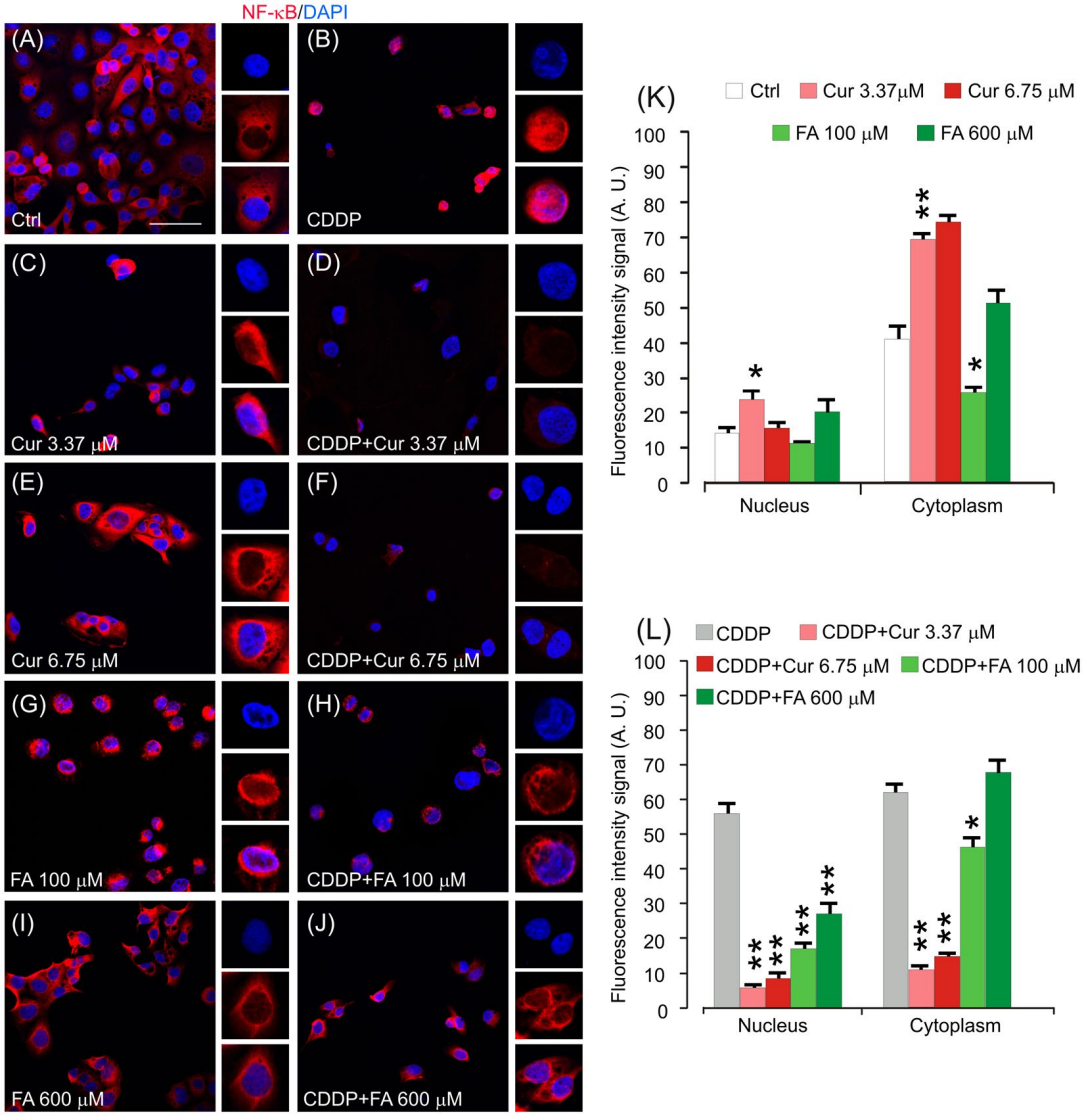
Curcumin decrease nuclear NF-κB expression in cancer cells. (**A**–**J**): Representative images of PECA-PJ15 immunolabelled with antibody against NF-κB (p65, red fluorescence) and stained with DAPI (blue fluorescence). High magnifications in boxes show nuclear *vs* cytosolic fluorescence localization. (**K**,**L**): Histograms (mean ± SEM) show fluorescence intensity signal quantification (A.U., arbitrary units) in the nucleus or in the cytoplasm of treated cells. Asterisks refers to significant differences compared to control condition in **K** and to cisplatin condition in **L** (*p < 0,05; **p < 0,01). Scale bar: 20 μm. Data are representative of three independent experiments, each count was performed on 10 fields randomly selected for each experimental condition and each time point.

To gain insight on inflammatory processes involved in cisplatin and polyphenol molecular mechanisms, immunofluorescence analysis for NF-κB (red fluorescence) on cancer cells was performed and results are shown in Fig. 6. In control condition, fluorescence signal was evident, mostly in the cytoplasm (Fig. 6A,K). Of note, cisplatin administration increased both nuclear and cytoplasmic fluorescence with respect to controls (Fig. 6B,L). Thus, cisplatin seems to increase inflammation in cancer cells, probably as a consequence of cell redox imbalance, similarly to what observed for Nrf-2 expression.

After curcumin treatment, NF-κB remained confined into the cytoplasm (Fig. 6C,E,K), whereas, in the combined exposure with cisplatin, curcumin decreased markedly both nuclear and cytoplasmic NF-κB expression with respect to cisplatin condition (Fig. 6D,F,L). Similarly to what observed in control condition, cell treated with FA both at 100 and 600 µM showed a primarily NF-κB cytoplasmic expression (Fig. 6G,I,K). When administered in conjunction with cisplatin, FA induced a decrease of nuclear fluorescence signal (Fig. 6H,L), although this effect was not comparable to what observed in cisplatin + curcumin treated specimens. Notably, in CDDP + FA 600 µM treated cells, NF-κB nuclear and cytoplasmic expression increased with respect to CDDP + FA 100 µM (Fig. 6J,L), indicating once again an hormetic effect of FA in cancer cells. Consistent with the curcumin effectiveness against ototoxicity, even in the *in vitro* model, it can be considered an efficient anti-inflammatory molecule that can counteract chemoresistant factors, such as NF-κB activation, promoting cisplatin anti-tumoral effects.

#### Polyphenols affects STAT-3 phosphorylation in cancer cells

**Figure 7:**
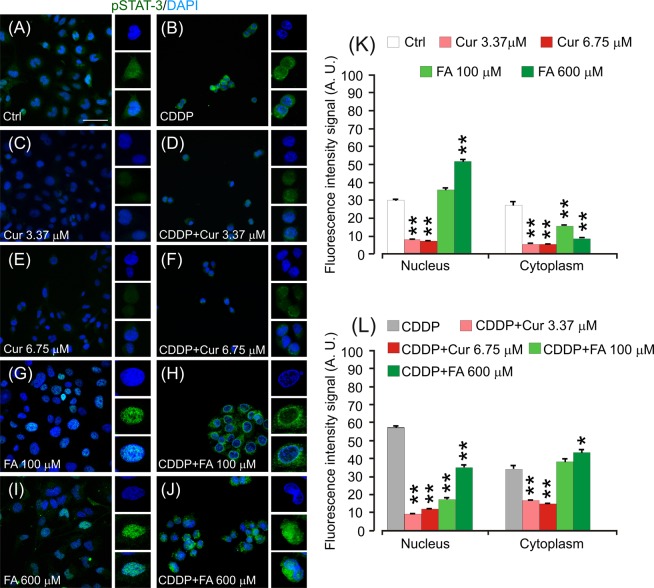
Curcumin inhibits cell proliferation targeting STAT-3 phosphorylation. (**A**–**J**): Representative images of PECA–PJ15 immunolabelled with antibody against pSTAT-3 (green fluorescence) and stained with DAPI (blue fluorescence). High magnifications in boxes show nuclear *vs* cytosolic fluorescence localization. (**K**,**L**): Histograms (mean ± SEM) show fluorescence intensity signal quantification (A.U., arbitrary units) in the nucleus or in the cytoplasm of treated cells. Asterisks refers to significant differences compared to control condition in **K** and to cisplatin condition in **L** (*p < 0,05; **p < 0,01). Scale bar: 20 μm. Data are representative of three independent experiments, each count was performed on 10 fields randomly selected for each experimental condition and each time point.

Moreover, to determine the effects of polyphenol treatment on tumor growth and on another chemosensitization factor, we analyzed the level of STAT-3 phosphorylation (pSTAT-3). Although in control specimens there was a clear pSTAT-3 labelling in the nucleus and in the cytoplasm (Fig. 7A,K), the fluorescence decreased markedly by increasing curcumin doses (Fig. 7C,E,K). In cisplatin-treated cells, pSTAT-3 labelling was evident in both cytoplasm and nucleus (Fig. 7B,L). In the combined treatment with curcumin and cisplatin, there was a marked decrease in STAT-3 phosphorylation with respect to cisplatin treatment alone (Fig. 7D,F,L). As regard FA treatment, pSTAT-3 nuclear signaling increased markedly by enhancing antioxidant doses (from 100 to 600 µM; Fig. 7G,I,K). When the antioxidant was administered in conjunction with cisplatin at the dose of 100 µM, we observed a change in fluorescence localization, which was mostly evident inside the cytoplasm (Fig. 7H,L). However, in the combined therapy with cisplatin and FA 600 µM, pSTAT-3 nuclear fluorescence signal increased (Fig. 7J,L). Taken together, these data indicate that curcumin and FA treatment show different ability to modulate STAT-3 signaling: when administered in conjunction with cisplatin, curcumin can counteract pSTAT-3 activation, acting as anti-proliferative molecule in a dose-dependent manner. On the other hand, FA showed a dose-dependent antioxidant effect and a biphasic behavior inducing pro-proliferative molecular targets. Notably, the increase of pSTAT-3 nuclear fluorescence observed in cells treated with cisplatin and FA 600 µM (Fig. 7J,L) is consistent with the enhanced cell survival observed in these cells with respect to cisplatin treated cells (Fig. 4D), indicating that high doses of FA can activate pro-proliferative mechanisms counteracting cisplatin cytotoxicity. Moreover, STAT-3 total expression did not differ in the different experimental groups, indicating that both cisplatin and polyphenols can target STAT-3 pathway by modulating protein phosphorylation but not protein expression as shown by total protein immunofluorescence (Supplementary Fig. S1).

#### Polyphenols modulate p53 phosphorylation in cancer cells

**Figure 8:**
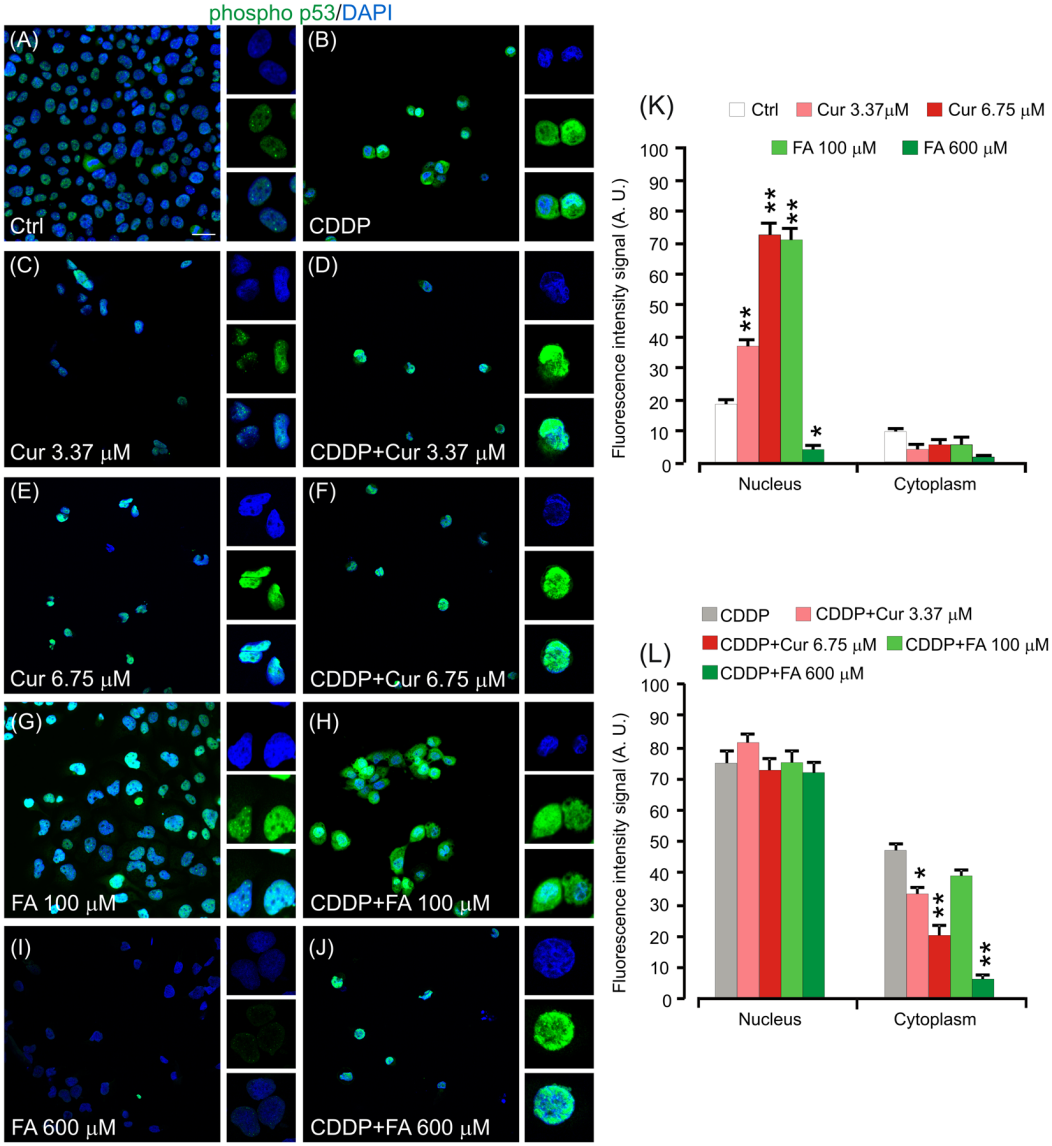
p53 phosphorylation is modulated by polyphenols in cancer cells. (**A**–**J**): Representative images of PECA–PJ15 immunolabelled with antibody against phospho (Ser 15) p53 (green fluorescence) and stained with DAPI (blue fluorescence). High magnifications in boxes show nuclear *vs* cytosolic fluorescence localization. (**K**,**L**): Histograms (mean ± SEM) show fluorescence intensity signal quantification (A.U., arbitrary units) in the nucleus or in the cytoplasm of treated cells. Asterisks refers to significant differences compared to control condition in **K** and to cisplatin condition in **L** (*p < 0,05; **p < 0,01). Scale bar: 20 μm. Data are representative of three independent experiments, each count was performed on 10 fields randomly selected for each experimental condition and each time point.

Prompted by the pro-apoptotic effects observed in cochlear cells, we characterized phospho p53 activation in PE/CA-PJ15 cells. Namely, the immunofluorescence analyses showed a slight nuclear expression of phospho p53 in control cells (Fig. 8A), confirmed by fluorescence signal quantification (Fig. 8K). After curcumin treatment, fluorescence increased markedly, specifically in the nucleus, whereas no signal was detected in the cytoplasm (Fig. 8C,E,K). FA showed different effects related to the concentrations: FA 100 µM induced increase of p53 phosphorylation similarly to curcumin (Fig. 8G,K). On the contrary, high FA concentrations (600 µM) induced a strong decrease of phospho p53 fluorescence signal that was almost absent both in the nucleus and in the cytoplasm (Fig. 8I,K). Tumor cells treated with cisplatin showed clearly a nuclear phospho p53 activation (Fig. 8B,L) that remained however unchanged in cells treated with both cisplatin and curcumin (Fig. 8D,F,L) or cisplatin and FA (Fig. 8H,J,L). Therefore, the adjuvant polyphenol treatment did not modify p53 phosphorylation induced by cisplatin, indicating that the pro-apoptotic activation of p53 is not the mechanism by which the adjuvant curcumin or FA can potentiate cisplatin cytotoxicity. Moreover, as shown in the supplementary figure 1, cisplatin and polyphenols treatments modulated similarly both p53 phosphorylation and its total expression: curcumin monotherapy or co-therapy with cisplatin induced a marked increase of p53 fluorescence, whereas high FA concentration (600 µM) induced a strong decrease of p53 fluorescence in monotherapy (Supplementary Fig. S1).

## Discussion

The major results indicated that polyphenols administrated in conjunction with cisplatin can exert both anti-oxidant and pro-oxidant effects; however, the polyphenolic compounds show different mechanisms of action depending on cell context and dosage. In fact, cisplatin in the cochlea causes an excessive ROS production resulting in redox imbalance and cell death similarly to what occurs in noise-induced hearing loss and presbycusis^21,34–37^. Here, we demonstrated that the increase of Nrf-2 nuclear translocation and HO-1 fluorescence in hair cells and spiral ganglion neurons (Fig. 2) after cisplatin treatment can be considered as an adaptive response to face oxidative cisplatin insult. Indeed, Nrf-2 is the master activator of the anti-oxidant defense program^38,39^ and among the enzymes upregulated by Nrf-2, HO-1 is one of the most representative stress response enzymes. HO-1 has prominent antioxidant and anti-inflammatory properties^40^. Additionally, cisplatin-induced ROS generation is a key contributor to inflammation and apoptosis in cochlear cells^18,41–43^, as demonstrated by NF-κB over-expression in cochlear structures in our *in vivo* model. NF-κB is another target for ROS^44^ and redox-dependent and independent mechanisms have been proposed to explain NF-κB activation, both of which, by using different pathways, have NF-κB as a downstream target^45^.

Furthermore, cisplatin administration increased p53 phosphorylation in the cochlea in agreement with several reports demonstrating the critical role of p53 accumulation and activation in cisplatin-induced hair cell death, since genetic deletion or pharmacological inhibition of p53 attenuates the loss of both types of hair cells, thus preserving hearing during cisplatin treatments^46,47^. Consistent with previous results^20,22,29^, we demonstrated that the two selected polyphenols attenuated hearing threshold elevation and in parallel, interacting with ROS signaling pathways, induced adaptive stress responses by up-regulating nuclear translocation of Nrf-2. Furthermore, we observed that the cisplatin-activated NF-κB expression was down-regulated only by curcumin in the principal cochlear structures (Fig. 3, upper panels), illustrating its anti-inflammatory effect. Also, both the anti-oxidant and anti-inflammatory effects of polyphenols led to the inhibition of p53 phosphorylation (Fig. 3, lower panels) and to decreased OHC death, the major hair cell population affected by cisplatin (Fig. 1). Thus, both polyphenols exhibit protective activities suggesting a promising approach for clinical translation, even if we demonstrated that the two major polyphenols act on different ways: antioxidant for FA and both anti-inflammatory and antioxidant for curcumin.

Conversely, in cancer cells polyphenols acted differently, both as anti-oxidant or pro-oxidant compounds. We found not only in cochlear cells but also in cisplatin-treated cancer cells that both cisplatin and dose-dependent polyphenol monotherapy increased Nrf-2 cytosolic fluorescence and nuclear translocation (see Fig. 5) indicating the activation of an adaptive stress protective response^48^. Interestingly in neoplastic cells, Nrf-2 up-regulation, correlated with cell proliferation and resistance to chemo- and radio-toxic effects, providing protection on cancer cells^49,50^. Loss of Nrf-2 or inhibition by siRNA result in increased cell death, cytotoxicity and apoptosis in response to cisplatin treatment compared with control cells^15,16^. Notably, in this and consistent with a previous study^18^ we report Nrf-2 segregation into the cytoplasm in the combinatory cisplatin/curcumin treatment, suggesting a cisplatin polyphenol sensitizatizing effect induced by curcumin inhibition of Nrf-2 translocation.

Here we also analyzed the inflammatory and pro-apoptotic responses in cisplatin-treated cancer cells and we found polyphenol down-regulation of the cisplatin-induced NF-κB and STAT-3 up-regulation and permissive p53 activity (Figs. 6, 7 and 8). Detailed analysis of NF-κB in inflammation associated malignancies has been provided^51,52^ and the link between inflammation and cancer has been confirmed by anti-inflammatory therapies that show efficacy in cancer prevention and treatment^53^. In fact, polyphenol caused down-regulation of cisplatin-induced NF-κB and STAT-3 up-regulation and a permissive p53 activity^54–56^. The other possible cross-talk is the one between NF-κB and Nrf-2. Indeed, suppression or inactivation of NF-κB-mediated transcriptional activity by Nrf-2 has been reported^57^ and, conversely, Nrf-2 can inhibit NF-κB pathway^58,59^. Claims have been provided for a role of NF-κB in cisplatin chemoresistance, increased aggressiveness, rapid tumorigenesis and drug resistance^50^.

The other possible Nrf-2 crosstalk concerns p53^60^. The p53 protein acts as a potent tumor suppressor^12^ and a major signaling pathway to regulate its expression is oxidative stress^4^. Under normal conditions, p53 is repressed by Murine double minute 2 (Mdm2), a transcriptional target of p53, that targets p53 for ubiquitination and proteasomal degradation serving as a negative feedback to restore homeostasis^61^. Loss of p53 function can be due to over expression of the protein Mdm2 and, notably, Mdm2 is shown to be a target of Nrf-2^62,63^. Evidence has been provided on the strong correlation between Nrf-2 activation and elevated Mdm2 expression in mouse and human malignant pancreatic ductal adenocarcinoma. Nrf-2 promotes the malignancy progression by inducing Mdm2 transcription, which abrogates the p53-imposed programming checkpoint favoring the onset and progression of many human malignancies^48^.

Last but not the least in relation to polyphenols, our data underlined the differential hormetic properties of polyphenols in normal *versus* cancer cells (see curves dose/response in the cochlea and cancer (Figs. 1 and 4). Hormesis is defined as the duality in response by a cell/individual in reply to an endogenous or exogenous impetus that spurs favorable effects at a low dose and harmful effects at higher measures^64^. In the cochlea we found a linear model of dose/response for FA and a U-shaped dose-response curve for curcumin. Cancer cells, however, responded to curcumin in the linear model and to FA in the U-shaped model. This duality of response may depend on dose, exposure times, pharmacokinetic properties of the molecules, environment of the target system and biological status of the target cell^64^. Although polyphenols reduce cisplatin ototoxicity, FA shows an unwanted tumor protecting effect that might restrict it clinical use.

In summary, even if many molecules have been designated as potential therapeutic targets for protection against cisplatin adverse effects, modulation of cisplatin-induced ototoxicity is still a doubtful balance between protection and tumor toxicity. Our data suggest that the polyphenol modulation of the adaptive stress response to cisplatin can be exerted by an interplay between the cellular defensive program and inflammation and the key players Nrf-2, NF-κB, STAT-3 and p53. Polyphenols possess powerful activities and their combined use with cisplatin may be a good therapeutic strategy to pursue in clinical trials of otoprotection; however, antioxidants such as FA should be cautioned for possible anti-cytotoxic activity in patients undegoing chemotherapy.

## Materials and Methods

### *In vivo* experiments

#### Animals

Male adult Wistar rats (UCSC Laboratories, 200–250 g) were used. The auditory function of each animal was tested for the presence of Preyer’s reflex. Experiments were performed on 96 animals, randomized and assigned to different experimental groups: control animals (Ctrl group; n = 6); cisplatin treated animals (CDDP group; n = 6); animals treated with cisplatin and curcumin (the latter used at doses of 100, 200 and 400 mg/kg; CDDP + Cur groups; n = 6 animals x group); animals treated with cisplatin and FA (the latter used at doses of 75, 150, 300 and 600 mg/kg; CDDP + FA groups, n = 6 animals x group); animals treated with different doses of curcumin (100, 200 and 400 mg/kg; Cur groups, n = 6 animals x group) and animals treated with FA (75, 150, 300 or 600 mg/kg, FA groups, n = 6 animals x group). Given that no significant differences have been found among Ctrl and Cur or FA groups, all data shown refer to Ctrl group. All animals were sacrificed under deep anesthesia (ketamine 70 mg/kg and medetomidine-dormitor 0.5 mg/kg) at day 5 after treatment onset. For the whole experimental period, the animals were housed 2 per cage at controlled temperature (22/23 °C) and constant humidity (60 ± 5%), under a 12-hour light/dark cycle, with food (Mucedola 4RF21, Italy) and water *ad libitum*. All efforts were made to minimize animal suffering and to reduce their number, in accordance with the European Community Council Directive of 24 November 1986 (86/609/EEC). The protocol was approved by Laboratory of Animal Care and Use Committee of the Catholic University, School of Medicine of Rome and by the Italian Department of Health (project identification code: prot. UCSC/14 U).

### Drug administration

Cisplatin (Cat. No. P4394, Sigma-Aldrich, St. Louis, MO, USA), diluted in sterile saline (1 mg/ml), was prepared freshly and protected by light. To facilitate drug dissolution, the solution was heated and stirred for a period of 20 minutes. Under deep anesthesia, a single cisplatin dose of 16 mg/kg^18,20^ was delivered intraperitoneally (i.p.) at a rate of 8 ml/h with an infusion pump (Axon Instruments, Foster City, CA, USA) over about 30 minutes. The animals were hyper-hydrated with saline solution (subcutaneous injection, 15 ml daily) to limit cisplatin side effects. As described previously^18^, curcumin (high purity, Cat. No. C7727, Sigma-Aldrich, St. Louis, MO, USA) was dissolved in dimethyl sulfoxide (DMSO). The diluted solution was prepared freshly daily and administered i.p. at three different doses (100, 200 and 400 mg/kg b.w.). FA (Cat. No. 12,870-8, Sigma-Aldrich, St. Louis, MO, USA) was diluted in DMSO, prepared freshly daily and administered i.p. at four different doses (75, 150, 300 and 600 mg/kg b.w.). Curcumin chosen concentrations were based on our preliminary evaluation of dose-response effects on cisplatin-induced hearing loss^18^. FA concentrations were extrapolated from previous *in vivo* studies in the guinea pig^29^. Curcumin or FA solution were injected 1 hour before cisplatin administration and once daily for the following 3 days, considering that previous studies demonstrated that this therapeutic window is effective in preventing cochlear exogenous insults^20–22^.

#### Auditory function evaluation

Auditory Brainstem Responses (ABRs) were measured at low (6 kHz), mid (12, 16, and 20 kHz), and high (24 and 32 kHz) frequencies. In all animals, ABRs were assessed bilaterally before treatment (day 0) to assure normal hearing and reassessed at all time points (3 and 5 days from treatment onset) to evaluate the effect of treatments on hearing. All animals were mildly anesthetized (ketamine, 35 mg/kg and medetomidine-dormitor, 0.25 mg/kg) and placed in the anechoic room. As described previously^18^, 3 stainless steel recording electrodes were subcutaneously inserted posterior to the tested pinna (active), vertex (reference) and contralateral pinna (ground). A PC-controlled TDT System 3 (Tucker-Davis Technologies, Alachua, FL, USA) data acquisition system with real-time digital signal processing was used for ABR recording and auditory stimulus generation. All procedures were performed in quiet, the TDT system for ABR recording is designed to minimize noise at every step. Tone bursts of pure tones from 6 to 32 kHz (1 ms rise/fall time, 10 ms total duration, 20/s repetition rate) were presented monaurally. Responses were filtered (0.3–3 kHz), digitized and averaged (across 512 discrete samples at each frequency-level combination, according to TDT User Guide). Threshold value was defined as the lowest stimulus level that yielded a repeatable waveform-based onset. The ABR data are expressed in terms of threshold shift that represents the difference between the pre and post-treatment values of each animal for each group.

#### Morphological analyses and cell viability

F–Actin staining was used to visualize the stereociliary arrays and cuticular plates of hair cells at day 5 in 6 cochleae/group. Surface preparations of the basilar membrane with the organ of Corti were processed. Briefly, the removed cochleae were fixed with 10% buffered Formalin for 4 h. After removal of the bony capsule and the lateral wall tissues, the epithelium of the organ of Corti was separated from the bony modiolus and dissected in half-turns in 0.1 M PBS under a dissecting microscope. Surface preparations of the organ of Corti were incubated with a solution containing ActinGreen 488 Ready Probes Reagent (Cat. No. R37110, Thermo Fisher, Waltham, MA, USA) in 0.1 M PBS for 30 min at room temperature protected from light. Positive cells were counted in segments of approximately 250 µm in length each along the basilar membrane. Hair cells were considered missing if both the stereocilia bundles and the cuticular plates were absent, and OHC loss was calculated as percentage with respect to controls. All morphologic observations were performed with the aid of the confocal laser scanning system (Nikon Ti-E, Confocal Head A1 MP, Tokyo, Japan).

#### Immunofluorescence analyses

Immunostainings were performed at day 5 in cochlear cryosections or in surface preparations of the organ of Corti, in order to assess and quantify the endogenous antioxidant response (Nrf-2/HO-1 pathway), inflammatory response (NF-κB p65) and apoptosis processes (p53 phosphorylation) in cisplatin-induced damage and to evaluate the effect of curcumin and FA supplementation. The cochleae (6/group) were quickly removed, and the samples were fixed with 4% paraformaldehyde in PBS at 4 °C and a pH 7.5. Next, the cochleae were decalcified for 15 days in EDTA (10% EDTA, changed daily), incubated for 48 h in sucrose (30%), embedded in OCT and cryosectioned at a thickness of 12 μm (Cryostat SLEE).

To perform immunofluorescence on surface preparations, after removal of the bony capsule and the lateral wall tissues, the epithelium of the organ of Corti was separated from the bony modiolus and dissected in half-turns in 0.1 M PBS under a dissecting microscope. The specimens were incubated with a blocking solution (1% BSA, 0.5% Triton X-100 and 10% normal goat serum in PBS 0.1 M) and then they were incubated overnight at 4 °C with a solution containing primary antibody against: HO-1 (1:100; Cat. No. ADI-SPA-895, Stressgen, Ann Arbor, MI, USA); Nrf-2 (1:100, Cat. No. ab137550, Abcam, Cambridge, UK); NF-κB (p65) (1:100, Cat. No. #8242, Cell Signaling Tech, Boston, MA USA); p53 (phospho S15, Cat. No. ab1431, Abcam, Cambridge, UK) diluted 1:100 in PBS. These antibodies cross-reacted with rat tissue. All specimens were incubated at room temperature for 2 h in labeled conjugated goat anti-rabbit and/or donkey anti-mouse secondary antibody (Alexa Fluor 488 and 546, IgG, Thermo Fisher) diluted 1:400 in 0.1 M PBS and DAPI stained (Cat. No. D1306, Thermo Fisher; 1:500 in 0.1 M PBS). Images were obtained with the confocal laser scanning system equipped with an Ar/ArKr laser (for 488 nm excitation) and HeNe laser (for 543 nm excitation). DAPI staining was imaged by two photon excitation (740 nm, < 140 fs, 90 MHz) performed with an ultrafast, tunable mode-locked Ti:sapphire laser. A semi-quantitative analysis of fluorescence signals in cochlear samples was quantified with ImageJ (version 1.51 s). Control experiments (negative controls not shown) were performed by omitting the primary antibody during processing of tissue randomly selected across experimental groups. Tissues from all groups were always processed together during the procedures to limit variability related to antibody penetration, incubation time, post-sectioning age, and condition of tissue.

### *In vitro* experiments

#### Cell line

We used PE/CA-PJ15 human oral squamous carcinoma cell line (European Collection of Cell Cultures) cultured in Iscove’s modified Dulbecco’s modified Eagle medium (DMEM, IMDM) supplemented with 10% fetal bovin serum (FBS) (Biochrom, Berlin, Germany), 1% penicillin/streptomycin (10000 U/ml/10000 μg/ml, Biochrom, Germany) and 1% of L-Glutamine 200 mM (Biochrom, Germany) at 37 °C, in an atmosphere of 95% oxygen and 5% CO_2_.

#### Drug administration

Cisplatin was dissolved in sterile saline and administered at a dose of 1.56 μM, dose used in previous work^18^. Cells were trypsinized and seeded on 13 mm cover slips in 24-well plates or in flask in quadruplicate and allowed to adhere overnight. Curcumin was dissolved in 0.5% DMSO and EtOH and sterile H_2_O (1:1); FA was dissolved in 0.5% DMSO. All manipulations were performed under subdued light. In agreement with previous report^18^ curcumin was administered at the doses of 0.5, 1, 3.37 and 6.75 µM, however data shown refer to the most effective doses (3.37 and 6.75 µM). To test the effect of FA, a dose-response curve was performed administering the antioxidant at 10, 100 and 600 µM.

#### Cell survival

In order to evaluate the effect of cisplatin, curcumin, FA and the combined drug exposures on cell survival, 1.0 × 10^4^ cells/glass were fixed with 4% paraformaldehyde for 15 minutes, washed twice in PBS and then incubated in a solution containing DAPI (1:1000 in PBS 0.1 M) and 0.1% Triton (in PBS 0.1 M) for 10 minutes light-protected and at room temperature. DAPI labeling was used to identify condensed cell nuclei. The sample were, then, washed in PBS and coverslipped with an antifade medium (ProLong Gold; Thermo Fisher). Cell count was performed on images acquired (10 × ) by a confocal laser scanning microscope (Nikon Ti-E, Confocal Head A1 MP, Japan) and processed with the aid of ImageJ Nucleus Counter Plugin (WCIF ImageJ, from http://www.uhnres.utoronto.ca/facilities/wcif/). Each count was based on 10 fields randomly selected for each of the experimental condition.

#### TUNEL assay

Apoptosis was evaluated in PE-CA/PJ15 cultures with the APO-BrdU TUNEL assay kit (Cat. No. A23210, Thermo Fisher) 48 h after treatment, according to the manufacturer’s instructions, as previously described^18^. Briefly, DNA strand breaks in apoptotic cells were labeled with BrdU by the use of terminal deoxynucleotide transferase. Apoptotic cells were identified immunocytochemically by means of anti-BrdU antibody labeling with Alexa Fluor 488 dye, and cell nuclei were identified by means of propidium iodide/RNAse staining. Cell count (TUNEL-positive cells) was performed on image acquired (10 × ) and processed with the aid of ImageJ Nucleus Counter Plugin. Each count was based on 10 fields randomly selected for each experimental condition. Results are reported as percentage of TUNEL-positive cells.

#### Immunofluorescence analyses

Cells were fixed with 4% paraformaldehyde for 15 minutes at room temperature, permeated with 0.1% Triton for 15 minutes prior to being blocked in 0.3% BSA for 20 min. Samples were then incubated with the following primary antibodies: anti-phospho-STAT-3 (Tyr705) (Cat. No. #9145, Cell Signaling Tech, Boston, MA, USA); anti-Nrf-2 (Abcam); anti-phospho p53 (phospho S15, Abcam) or anti-NF-κB (Cell Signaling Tech.) for 3 h in 0.3% BSA in PBS. At the end of incubation, all samples were washed twice in PBS and incubated at room temperature for 90 minutes, light-protected, with secondary antibody goat anti-rabbit (Alexa Fluor 488, Thermo Fisher) or donkey anti-mouse (Alexa Fluor 546, Thermo Fisher) diluted 1:1000 in PBS. Moreover, cell nuclei were counterstained with DAPI (Thermo Fisher; 1:1000 in PBS) for 10 min at room temperature, light-protected. Then, the samples were coverslipped with an antifade medium (ProLong Gold; Thermo Fisher). Images of immunolabeled specimens (40 × ) were taken by the confocal laser scanning microscope (Nikon, Japan). Immunofluorescence was performed 24, 48 and 72 h after treatment. However, given that no significant differences were observed among the three time points, only the results at 24 h are discussed. Control experiments (negative controls not shown) were performed by omitting the primary antibody during processing of tissue randomly selected across experimental groups. Samples were always processed together during the procedures to limit variability related to antibody penetration or incubation time. To perform semi-quantitative analysis of fluorescence signals, fluorescence intensity was quantified with ImageJ (version 1.51 s) on N = 10 field randomly selected from each experimental group. Moreover, immunofluorescence analyses for total STAT-3 and total p53 expression was performed and a ratio of fluorescence intensity signals of phospho STAT-3 and phospho p53 *versus* total STAT-3 and total p53 respectively was calculated, as detailed in Supplementary Materials and Methods.

#### Statistical analyses

Results are presented as means ± standard error of mean (S.E.M.) and differences were assessed using variance analysis (ANOVAs). Power analysis was performed to determine the sample size to provide a statistical power of 80% at an α level of 0.05. As regards cell survival a two-way ANOVA was performed (group × time point). To analyze TUNEL-positive cells, one way ANOVA was performed (between factor: group). ABR data were evaluated by three-way ANOVA (ABR: group × frequency × time point). Cochleogram data were analyzed by two-way ANOVA (group × cochlear turn). *Post-hoc* comparisons were assessed using Tukey’s test (Statistica, Statsoft, Tulsa, OK, USA); *p* < 0.05 was considered significant.

## Supplementary information

Supplementary Informations.

## Data Availability

The data that support the findings of this study are available from the corresponding authors on reasonable request.
