# Publisher Correction: The dual role of curcumin and ferulic acid in counteracting chemoresistance and cisplatin-induced ototoxicity

**DOI:** 10.1038/s41598-020-70073-3

**Published:** 2020-09-30

**Authors:** Fabiola Paciello, Anna Rita Fetoni, Daniele Mezzogori, Rolando Rolesi, Antonella Di Pino, Gaetano Paludetti, Claudio Grassi, Diana Troiani

**Affiliations:** 1 Department of Neuroscience, Università Cattolica del Sacro Cuore, Roma, Italia; 2 Fondazione Policlinico Universitario A. Gemelli IRCCS, Roma, Italia; 3 Department of Head and Neck Surgery, Università Cattolica del Sacro Cuore, Roma, Italia

Correction to: *Scientific Reports* 10.1038/s41598-020-57965-0, published online 23 January 2020.

In the original version of this Article, the author Anna Rita Fetoni was incorrectly indexed. This error has now been corrected.

