# Supplementary material for: The dual role of curcumin and ferulic acid in counteracting chemoresistance and cisplatin-induced ototoxicity
Source: Sci Rep. 2020 Jan 23;10:1063. doi: 10.1038/s41598-020-57965-0 (PMC6978317; doi:10.1038/s41598-020-57965-0)
Supplement: Supplementary file 1 — Supplementary Informations. [file 41598_2020_57965_MOESM1_ESM.pdf]

The dual role of curcumin and ferulic acid in counteracting chemoresistance and cisplatin-induced ototoxicity

Fabiola Paciello[1,2], Anna Rita Fetoni,[2,3]*, Daniele Mezzogori[1], Rolando Rolesi[2], Antonella Di Pino[3], Gaetano Paludetti[2,3§], Claudio Grassi[1,2§] and Diana Troiani[1§.]

[1]Department of Neuroscience, Università Cattolica del Sacro Cuore, Roma, Italia;

[2]Fondazione Policlinico Universitario A. Gemelli IRCCS, Roma, Italia;

[3]Institute of Otolaryngology, Università Cattolica del Sacro Cuore, Roma, Italia.

Supplementary Materials and Methods

STAT-3 and p53 immunofluorescence analyses in cancer cells.

Cells were fixed with 4% paraformaldehyde for 15 minutes at room temperature, permeated with 0.1% Triton for 15 minutes prior to being blocked in 0.3% BSA for 20 min. Samples were then incubated with the following primary antibodies: anti- STAT-3 (Cat. No. #9139, Cell Signaling Tech, Boston, MA, USA); anti-p53 (Cat. No. #2524, Cell Signaling Tech) for 3 h in 0.3% BSA in PBS. At the end of incubation, all samples were washed twice in PBS and incubated at room temperature for 90 minutes, light-protected, with secondary donkey anti-mouse antibody (Alexa Fluor 546, Thermo Fisher) diluted 1:1000 in PBS. Moreover, cell nuclei were counterstained with DAPI (Thermo Fisher; 1:1000 in PBS) for 10 min at room temperature, light-protected. Then, the samples were coverslipped with an antifade medium (ProLong Gold; Thermo Fisher). Images of immunolabeled specimens (40×) were taken by the confocal laser scanning microscope (Nikon, Japan). Control experiments (negative controls not shown) were performed by omitting the primary antibody during processing of tissue randomly selected across experimental groups. Samples were always processed together during the procedures to limit variability related to antibody penetration or incubation time. To perform semi-quantitative analysis of fluorescence signals, fluorescence intensity was quantified with ImageJ (version 1.51s) on N=10 field randomly selected from each experimental group.

Supplementary Figure 1

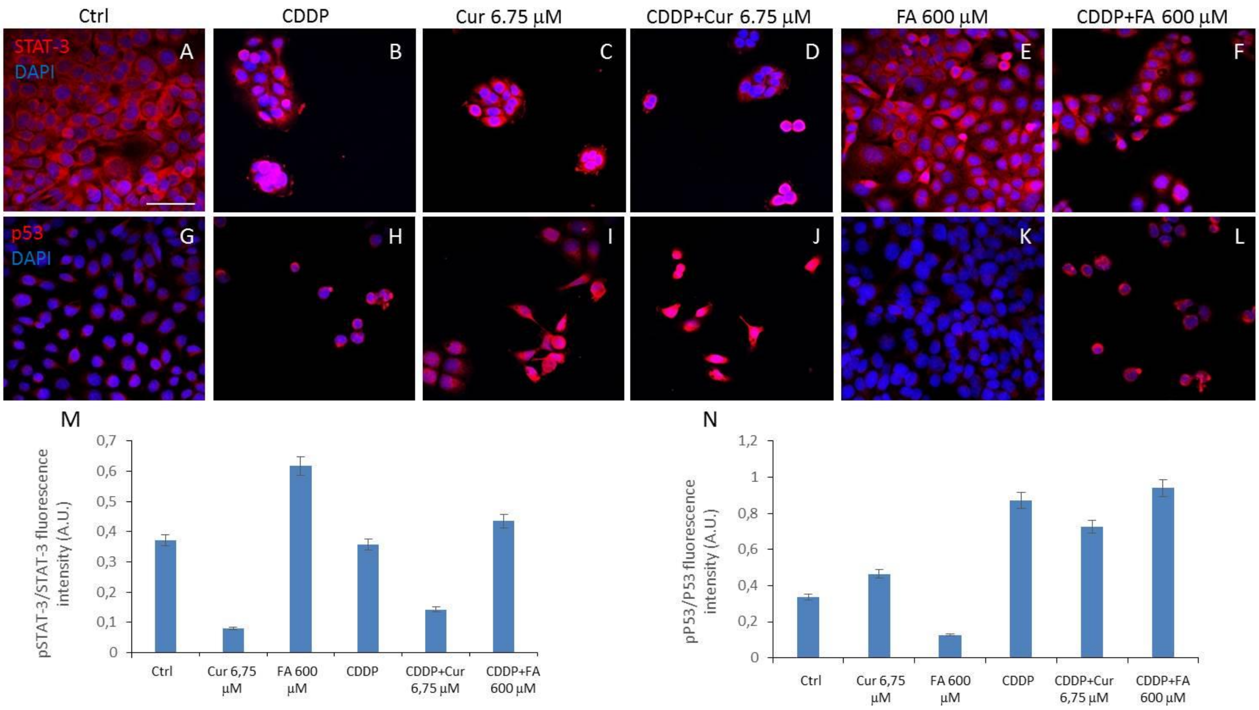

Figure S1: Total STAT-3 and p53 expressions in cancer cells. A-L: Representative images of PECA-PJ15 immunolabelled with antibody against STAT-3 (A-F) or p53 (G-L) and stained with DAPI (blue fluorescence). M,N: Histograms (mean ± SEM) show the ratio between phospho STAT-3 and total STAT-3 (M) or phospho p53 and total p53 (N) fluorescence intensity signal (A.U., arbitrary units). Scale bar: 50 μm.
